# Sustainability of healthcare professionals’ adherence to clinical practice guidelines in primary care

**DOI:** 10.1186/s12875-022-01641-x

**Published:** 2022-03-01

**Authors:** Xian-Liang Liu, Tao Wang, Jing-Yu Tan, Simon Stewart, Raymond J. Chan, Sabina Eliseeva, Mary Janice Polotan, Isabella Zhao

**Affiliations:** 1 College of Nursing and Midwifery, Charles Darwin University, 410 Ann Street, Brisbane, QLD 4000 Australia; 2 Torrens University Australia, Wakefield Campus, Adelaide, SA 5000 Australia; 3 University of Glasgow, Glasgow, Scotland, UK; 4 Caring Futures Institute, College of Nursing and Health Sciences, Flinders University, Bedford Park, SA 5042 Australia; 5 Thornlands General Practice, 51 Island Outlook Ave Thornlands, Redland, QLD 4164 Australia; 6 Cancer & Palliative Care Outcomes Centre, Faculty of Health, Queensland University of Technology, 60 Musk Avenue, Kelvin Grove, QLD 4059 Australia

**Keywords:** Sustainability, Healthcare professionals, Adherence, Clinical practice guidelines, Primary care

## Abstract

**Background:**

Sustainability of adherence to clinical practice guidelines (CPGs) represents an important indicator of the successful implementation in the primary care setting.

**Aim:**

To explore the sustainability of primary care providers’ adherence to CPGs after receiving planned guideline implementation strategies, activities, or programmes.

**Methods:**

Cochrane Central Register of Controlled Trials (CENTRAL); Cumulative Index to Nursing and Allied Health Literature (CINAHL); EMBase; Joanna Briggs Institute; Journals@Ovid; Medline; PsycoINFO; PubMed, and Web of Science were searched from January 2000 through May 2021 to identify relevant studies. Studies evaluating the sustainability of primary care providers’ (PCPs’) adherence to CPGs in primary care after any planned guideline implementation strategies, activities, or programmes were included. Two reviewers extracted data from the included studies and assessed methodological quality independently. Narrative synthesis of the findings was conducted.

**Results:**

Eleven studies were included. These studies evaluated the sustainability of adherence to CPGs related to drug prescribing, disease management, cancer screening, and hand hygiene in primary care. Educational outreach visits, teaching sessions, reminders, audit and feedback, and printed materials were utilized in the included studies as guideline implementation strategies. None of the included studies utilized purpose-designed measurements to evaluate the extent of sustainability. Three studies showed positive sustainability results, three studies showed mixed sustainability results, and four studies reported no significant changes in the sustainability of adherence to CPGs. Overall, it was difficult to quantify the extent to which CPG-based healthcare behaviours were fully sustained based on the variety of results reported in the included studies.

**Conclusion:**

Current guideline implementation strategies may potentially improve the sustainability of PCPs’ adherence to CPGs. However, the literature reveals a limited body of evidence for any given guideline implementation strategy. Further research, including the development of a validated purpose-designed sustainability tool, is required to address this important clinical issue.

**Trial registration:**

The study protocol has been registered at PROSPERO (No. CRD42021259748).

**Supplementary Information:**

The online version contains supplementary material available at 10.1186/s12875-022-01641-x.

## Contributions to the literature

- Current guideline implementation strategies may potentially improve the sustainability of primary care providers’ adherence to clinical practice guidelines;
- No structured evaluation methods or purpose-designed tools were utilized to assess the healthcare professionals’ sustainability levels.
- Maximum effort should be taken to ensure the long-term continuation of the implementation by planning the sustainability of adherence to clinical practice guidelines carefully and adopting a multipronged strategic approach.

## Background

Clinical practice guidelines (CPGs) are evidence-based and systematically developed summaries and recommendations to assist healthcare professionals and patients in the process of healthcare decision-making [1]. CPGs can facilitate translation of up-to-date scientific research knowledge into practice and optimise care practices and outcomes for patients and their families [2]. When CPGs are adhered to, healthcare processes structures outcomes improve in primary care settings [3–5]. However, previous studies have shown that non-compliance of CPGs is as high as 70% in healthcare and occurs across most disciplines [6], including primary care [7]. Even in situations where there is CPG uptake, healthcare professionals may return to established clinical routine and practices, demonstrating and, therefore, have difficulty sustaining the successful implementation of CPGs in practice over a long period of time [6]. Strategies and activities undertaken beyond implementation to sustain CPG uptake are often inadequate [8]. Therefore, although CPGs are available and accessible to all healthcare professionals in primary care, the quality of healthcare services delivered to patients continues to vary [9, 10].

Implementation science refers to “…the scientific study of methods to promote the systematic uptake of research findings and other evidence-based practice into routine practice and, hence, to improve the quality and effectiveness of health services. (page 1)” [11] Implementation science tests the contextual factors affecting uptake and use of a clinical innovation, including sustainability, feasibility and fidelity [12]. Sustainability is a key outcome and priority quality indicator in implementation science. It refers to the extent to which a successful practice or programme is maintained as a clinical routine until it reaches obsolescence [13]. “Capability of being maintained at a certain rate or level (p. 1580)” is the simplest definition of sustainability [14]. Sustainability is often considered a result of maintaining health benefits or activities (e.g., cancer medication use and education) [13], and outcomes related to the implementation process (e.g., increased rates in continued use of the evidence-based innovations) [15]. The successful implementation of and adherence to CPGs in primary care is undeniably difficult. Several studies have explored the sustainability of innovations in primary care and reported that the innovations were not maintained after project funding had ended [16]. Overall, the sustainability of programmes were classified as either poor [17] or optimistic (sustainability score of > 55/100) [18]. The sustainability of the implementation of CPGs may require a well-structured process to ensure policies become fully integrated. Important determinants of success or failure in this context include the implementation process, staff, and organizational factors [19, 20]. One previous review aimed to evaluate the sustainability of healthcare professionals’ adherence to CPGs in all healthcare settings. It reported that structured approaches and methods for sustainability evaluations were lacking [8]. Further, only three of the 14 included studies in the previous systematic review focused on primary care, with mixed sustainability results identified in that setting [21–23]. Therefore, no definitive conclusion could be drawn about the effects of any of the implementation strategies for the sustainability of primary care professionals’ adherence to CPGs [8]; this being one of the least studied and understood issues in implementation research.

Within the above context, the aim of this literature analysis was to explore the sustainability of healthcare professionals’ adherence to CPGs after receiving planned guideline implementation strategies, activities, or programmes in primary care. Specifically, this review explored (1) the effectiveness of different activities and programmes that targeted healthcare professionals in primary care to improve the sustainability of adherence to CPGs; (2) the current sustainability of healthcare professionals’ adherence to CPGs in primary care; (3) the evaluation methods for the sustainability of adherence to CPGs in primary care; and (4) directions for increasing the sustainability of healthcare professionals’ adherence to CPGs in primary care in future.

## Methods

The Preferred Reporting Items for Systematic Reviews and Meta-Analyses (PRISMA) checklist was utilized in determining the information and process required for this literature analysis [24]. The study protocol has been registered at PROSPERO (No. CRD42021259748).

### Data sources

#### Electronic database search

An electronic database search was performed in nine databases to locate eligible publications from January 2000 through May 2021, and the search was limited to the English language only. The research team developed tailored search strategies in consultation with a university librarian. The databases were: Cochrane Central Register of Controlled Trials (CENTRAL); Cumulative Index to Nursing and Allied Health Literature (CINAHL); EMBase; Joanna Briggs Institute; Journals@Ovid; Medline; PsycoINFO; PubMed, and Web of Science. The following registers for ongoing or completed trials were also searched: ClinicalTrials.gov (https://clinicaltrials.gov/) and the Australian New Zealand Clinical Trials Registry (http://www.anzctr.org.au/). Moreover, the National Guideline Clearinghouse (NGC), the National Institute for Health and Care Excellence (NICE) and Turning Research Into Practice (TRIP) were also searched as secondary resources. The electronic database search strategies are reported in Additional file 2, and MESH terms and keywords were utilized in the database search (see Table 1).

**Table 1:** MESH terms and keywords

	Mesh Terms	Entry Terms, Key Words or Free Words
**Sustainability**	*“Guideline Adherence”*	“Sustainab*” or “Sustain*” or “Adherence*” or “Compliance*” or “Maintenance*”
**Clinical practice guidelines**	*“Implementation Science” or “Guidelines” or “Consensus”*	“Guidelin*” or “Pathway*” or “Evidence-based Recommendation*” or “expert opinion*”
**Primary care**	*“Primary Health Care” or “Physicians, Primary Care” or “Primary Care Nursing” or “Community Health Services”*	“Primary Care” or “Primary Healthcare” or “General Pract*” or “Practice Nurs*” or “Community Healthcare”
**Health professionals**	“*Health Personnel” or “General Practitioner” or “Nurses, Community Health”*	“Allied Health Provider*” or “Allied Health Professional*” or “Community Healthcare Worker*” or “Healthcare Professional*” or “Health Professional*” or “Therapist*” or “Dietitian*” or “Paramedics”

#### Reference list search

The reference lists of the included studies were searched using the ISI Web of Science for publications, which cites included studies.

### Inclusion criteria

1. Types of studies: Randomized controlled trials (RCTs), non-randomized studies, and before-after controlled studies.
2. Types of participants: Healthcare professionals working in primary care settings, including general practitioners (GPs), practice nurses, allied health providers, therapists (e.g., physiotherapists, occupational therapists, music therapists, and speech and language therapists), dietitians, paramedics, and community healthcare workers. Primary care was defined as “the provision of integrated, accessible health care services by clinicians who are accountable for addressing a large majority of personal health care needs, developing a sustained partnership with patients, and practicing in the context of family and community” (p. 192) [25].
3. Types of interventions: Any planned strategies, activities, or programmes (e.g., professional, organizational, and financial programmes) as part of a guideline implementation project that facilitated the sustainability of healthcare professionals’ adherence to CPGs in primary care. The sustainability of healthcare professionals’ adherence to CPGs was one of the study outcomes.
4. Types of comparators for controls: Usual conventional practice in primary care or only passively received guidelines without any planned guideline implementation strategies, activities, or programmes.
5. Types of outcome measures: Any objective and subjective measure of the sustainability of healthcare professionals’ adherence to CPGs (e.g., how long a CPG was sustained, follow-up sustainability assessment, and self-reported performance in the sustainability period) were included. Sustainability was defined as “the continued use of program components and activities for the continued achievement of desirable program and population outcomes” (p 2060) [26]. Therefore, multiple sustainability measuring methods were included even though the publications or reports did not mention the “sustainability” in the text, for example, quality of drug prescribing and cancer screening rates in accordance with the CPGs. The assessment of sustainability required its successful implementation in part of a practice, programme, or service that was then sustained for at least six months follow-up after the completion of the CPGs implementation strategies.

### Assessment of eligibility

Based on the inclusion criteria, all the retrieved articles’ titles and abstracts were assessed by the reviewers during the search process. After reading the titles and abstracts of all the retrieved articles, all duplicated articles were excluded by EndNote version X9 (Clarivate Analytics, London, United Kingdom). Two reviewers (XLL and TW) independently read the titles and abstracts of all the potentially relevant studies that were identified by the initial broad literature search. If information from the titles and abstracts was not clear, the full texts of the papers were retrieved for further assessment. Decisions to include a study in the review were made by two reviewers (XLL and TW) after appraisal of the full texts of all retrieved articles. Any doubts during this process were settled by discussion and, if necessary, with a third reviewer (JYT). All excluded full-text articles were given specific reasons why they were excluded, and a list of excluded papers was summarized.

### Assessment of the risk of bias in the included studies

The methodological quality and risk of bias were evaluated for each of the included studies using the Effective Practice and Organization of Care (EPOC) “risk of bias” tool [27]. The risk of bias of RCTs, non-randomized studies, and controlled before-after studies were assessed using nine risk of bias criteria related to randomization, allocation concealment, baseline outcome measures, baseline characteristics, outcome assessment, incomplete data, contamination, selective reporting, and other risk of bias [27, 28]. The EPOC “risk of bias” tool provides instructions for making decisions about the nine specific criteria as high, unclear, or low risk (see Supplemental file 1) [27].

If the details were not available in the articles, additional information was collected by contacting the corresponding authors of the relevant articles or reviewing their previously published protocols and articles. Two reviewers (XLL and TW) evaluated the risk of bias of the included studies independently, and any doubts during this process were settled by consultation or discussion with a third reviewer (JYT).

### Data extraction

Data from included studies were extracted independently by two reviewers (XLL and TW). The reviewers utilized a predefined data extraction form to extract data from each included article. The study designs, research settings, participant demographics, guideline implementation strategies/activities/programmes, comparisons, sustainability outcomes, and sustainability measurements were extracted. For missing or unclear information on the details of the included studies, attempts were made to contact the authors of the included studies or review their previously published protocols and articles to obtain additional information, if possible.

### Data analysis

The variability in guideline implementation strategies, CPGs, and the sustainability outcomes precluded a meta-analysis. Descriptive analysis was used for the synthesis of findings, including a summary of the characteristics of the sustainability assessments and descriptions of the level of sustainability of healthcare professionals’ adherence to CPGs in primary care after planned activities or programmes. Narrative subgroup analysis was conducted based on different sustainability outcomes, including sustainability of drug prescribing improvement, chronic disease management, cancer screening and hand hygiene practice. The sustainability results from the included studies were summarized descriptively for comparisons between the planned activities and programmes.

## Results

### Selection of studies

**Fig. 1:**
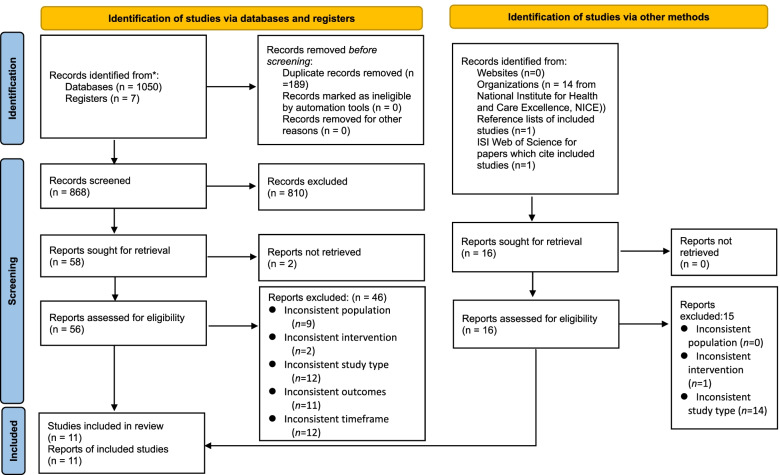
PRISMA flow diagram for search results

Figure 1 illustrates the number of publications identified at each step and the reasons for their exclusion. Overall, the systematic database search yielded 1057 potential records. Based on the applied eligibility criteria, 58 records appeared related to the research topic and required a further assessment of their full texts. However, 48 records were subsequently excluded at this step. In addition, this review also incorporated a search of key organizations (e.g., TRIP, NGC, and NICE), the reference lists of the included studies, and the ISI Web of Science for publications. The organization searches yielded 16 potential records, 15 of which were excluded because they did not meet the inclusion criteria. As a result, a total of 11 studies were included in this literature analysis [21–23, 29–36].

### Characteristics of the included studies

Table 2 provides a summary of the 11 included studies. These comprise nine RCTs [22, 23, 30–36], one non-randomized controlled trial [29], and one before-after study [21]. The included studies were conducted in the United Kingdom [21, 22, 30], the Netherlands [33, 36], the United States [23, 32], Belgium [29], Portugal [31], Japan [34], and Spain [35]. Six studies reported the funding sources of their studies, such as Diabetes UK, the National Health Institute, and Pfizer [22, 23, 30, 31, 33, 36]. Eight studies comprised a total of 1705 healthcare professionals who completed the intervention and follow-up, while three of the included studies only reported the number of primary care centres that participated in the studies (*n* = 84 [33], *n* = 28 [22], and *n* = 1 [21], respectively). Of the eight that reported number of participants, the average sample size of the included studies was 213 (range: 36–371). Most of the participants were GPs, and one included study recruited healthcare professionals from seven disciplines, including assistants in nursing, dental hygienists, GPs, midwives, nurses, odontostomatologists, and paediatricians [35]. The mean follow-up period was 16.1 months, ranging from six [29, 35] to 36 months [21].

**Table 2:** Characteristics of included studies

First author, year, setting, funding source	Study design	Participants (***n***)	Healthcare professional type	Intervention		Sustainability timeframe	Sustainability outcomes
				Intervention group(s)	Control group(s)		
Spitaels D, 2019, Belgium, NA [29]	Non-randomized and controlled intervention study	Participated: Intervention group: 426 GPs Control group: 798 GPs Completed the outcome questionnaire: Intervention group: 73 GPs Control group: 103 GPs	GPs	A 20-min, face-to-face educational outreach visit, with composed of two-components: face to face meeting about the evidence-based knee osteoarthritis management and a printed leaflet was provided for the GPs at the end of the educational visit.	Not visited by academic detailers.	Six months post-intervention	**N:** (1) Prescription of physical therapy: no significant change between the control and intervention group; (2) Quality indicator adherence: no significant change between the control and intervention group.
Presseau J, 2018, England, Diabetes UK [30]	Two-arm cluster RCT	Randomized: Intervention group: 22 primary care practices (153 GPs, nurses, and HCAs) Control group: 22 primary care practices (172 GPs, nurses, and HCAs) Completed: Intervention group: 22 primary care practices Control group: 20 primary care practices	GPs, nurses and healthcare assistants	Implementation intervention used behaviour change theory (e.g., SCT, Health Action Process Approach) and behaviour change techniques to develop the intervention, and involved outreach visits, to allow healthcare professionals to dedicate 90 min together to discuss the targeted healthcare behaviors, provided with materials to pre-identify barriers and solutions, short videos using practice-based examples, common barriers and possible solutions.	No intervention was provided and provided materials to control group at the end of the study.	12 months follow-up	**N:** Six guideline-recommended behaviors: no significant improvement: Electronic medical records: blood pressure and glycaemic control prescribing, physical activity and nutrition advice: no significant differences between the two groups were found; Patient survey: diabetes health education and foot examination
Pinto D, 2018, Portugal, National Health Institute [31]	Parallel, open, superiority, cluster RCT	Intervention group: 19 clusters with 120 participating physicians Control group: 19 clusters with 119 participating physicians	Family physicians	During a 6-month period, 3 educational outreach visits, 3 guidelines were chosen for dissemination, each educational outreach visit was focused on one guideline, last from 15 to 20 min. Each visit distributed a point of care summary, and a brochure was utilized as a visual aid.	Passive dissemination: by the publication on website.	18 months after the intervention.	**N:** Prescribed COX-2 inhibitors and the proportion of omeprazole: no statistically significant differences were found.
Wang H-YJ, 2018, USA, NA [32]	Two-arm cluster RCT	Randomized: Intervention group:13 practices, 246 patients Control group: 12 practices, 233 patients Completed: Intervention group: 13 practices, 195 patients Control group: 12 practices, 176 patients	Primary care physicians	Used social cognitive theory (SCT) to develop the intervention. Consisted of three components: (1) a printed communication guide, (2) 2 in-office, structured training with patients, each training lasted approximately 45 min and the second training session was 4 to 6 months later after the first session, and (3) auxiliary materials.	No intervention materials except the local free/low-cost screening information sheet	12-month follow-up	**N:** Patients’ self-reported receipt of routine colorectal cancer screening: small, non-significant effects.
Trietsch J, 2017, Netherlands, ZonMw [36]	Two-arm cluster RCT	Arm A: 10 LQICs, 39 practices (86 GPs) Arm B: 11 LQICs, 49 practices (122 GPs)	GPs	Audit and feedback with peer review in LQICs: each GP received performance feedback report, each group planned two meetings for each clinical topic (three topics in total from five different topics), test ordering and prescribing meeting, a total of six meetings. Each meeting: 90 to 120 min. Feedback reports were generated from diagnostic tests and prescriptions.	Same with Arm A on different topics (three topics in total from five different topics).	9 months after meetings	**N:** Tests ordered volumes, and drugs prescribed/practice/1000 patients/6 months: no statistically significant differences were found.
van der Velden AW, 2017, Netherlands, ZonMw [33]	Open, pragmatic, cluster RCT	Randomized: Antibiotic intervention group: 45 practices Control group: 41 practices Completed (second year): Antibiotic intervention group: 44 practices Control group: 40 practices	GPs	Multifaceted program aims to improve antibiotic use for RTIs was consisted of GP education, audit/feedback and patient information, two 4-week registration of RITs; Educational meeting: A meeting discussed the antibiotic prescribing guidelines and antibiotic-related problems with all GPs working in that primary care center in one session (60 to 90 min); An improvement plan was defined on optimize antibiotic prescribing after the educational meeting. Patient booklets: symptomatic treatment, natural course and alarm symptoms. Feedback: all antibiotics dispensed in the year after the GPs meeting.	Usual practices	Two years after the program	**P:** Overprescribing, non-first choice prescribing and underprescribing for RTIs: sustainably improved antibiotic prescribing, significant differences between the control and intervention group.
Noto H, 2016, Japan, NA [34]	Open cluster-RCT	Randomized: Intervention group: 22 PCPs Control group: 20 PCPs Completed: Intervention group: 21 PCPs/230 patients Control group: 15 PCPs/181 patients	PCPs	Received a copy of the *Diabetes Treatment Guide*, a copy of ‘The Manual and a 30-min seminar regarding ‘The Manual” and the updated copies were disseminated later.	Received a copy of the *Diabetes Treatment Guide*.	1-year follow-up period	**M:** Retinopathy evaluation (once annually), and measurements of urinary albumin excretion (every half year, significantly higher in the intervention group compared with the control group, p = 0.016) and serum creatinine (every half year).
Gerber JS, 2014, USA, Pfizer [23]	Cluster RCT	180 healthcare professionals Intervention group: 9 practices Control group: 9 practices	Pediatric primary care practices	(1) healthcare professional education, a 1-h review of current guidelines in prescribing, and (2) audit and feedback of antibiotic prescribing.	No intervention	18 months follow up	**P:** Broad-spectrum antibiotic prescribing: sustainably improved antibiotic prescribing.
Martín-Madrazo C, 2012, Spain, NA [35]	Cluster, parallel RCT	Randomized: Intervention group: 104 healthcare professionals (5 centers) Control group: 110 healthcare professionals (5 centers) Completed follow up: Intervention group: 84 healthcare professionals ((5 centers) Control group: 86 healthcare professionals (6 centers)	auxiliary nurses, dental hygienists, GPs, nurses, pediatricians, midwives and odontostomatologists.	Teaching sessions (Training of healthcare workers) were provided by two nurses within 1 month: 4 sessions of 50 min each for each primary care center on implementation of hydroalcoholic preparations, a video demonstrated the hand hygiene technique (6 steps), each consultation office placed hydroalcoholic solutions, and reminder poster on the walls were placed at key locations (e.g., consultation office, emergency room and waiting room).	No intervention	6 months follow up	**P:** Hand hygiene compliance level: statistically significant differences were found, *p* = 0.001.
Enriquez-Puga A, 2009, England, Eli Lilly [22]	RCT	Antidepressant prescribing group: 14 general practices Antibiotic prescribing group: 14 general practices	General practices	Antidepressant prescribing: Educational outreach visits: first visit: lasted 20 to 40 min, group interactive discussion on the appropriate prescribing, barriers to change, and best solution to overcome them. A second visit: feedback on prescribing and clarify outstanding issues	Antibiotic prescribing: same with the antibiotic prescribing group.	Two years after the first educational outreach visit	**M:** Number of items prescribed for amoxicillin with clavulanic acid (co-amoxiclav); average daily quantities for lofepramine; fluoxetine antidepressants and quinolone antibiotics: prescribing of lofepramine (a tricyclic antidepressant) had increased, *p* < 0.001.
Cates CJ 2009, England, NA [21]	Before–after study	One practice (Manor View) and a nearby control practice (Attenborough)	General practices	Evidence-based printed handout for parents and provide a deferred antibiotic prescription (with not to offer the antibiotics immediately advice).	Usual practices	Three years	**M:** Prescribing levels of amoxicillin suspension: fell significantly more than national levels.

*GPs* general practitioners, *RCT* randomized controlled trial, *HCAs* healthcare assistants, *LQICs* local quality improvement collaboratives, *RTIs* respiratory tract and ear infections, *SCT* social cognitive theory, *PCP* primary care physician, *NA* not applicable

care physician; NA, not applicable

Summary of the sustainability outcomes:

P: positive sustainability results

M: mixed sustainability results

N: no significant change

The CPGs of focus widely varied. None of the included studies implemented the same CPG. Six studies targeted adherence to guidelines related to general practice drug prescribing [21–23, 31, 33, 36], whereas three studies aimed to improve adherence to guidelines related to disease management [29, 30, 34] and one study each targeted improved cancer screening [32] and hand hygiene practice [35]. For the control groups, the same strategy was utilized for both the intervention group and the control group for different clinical topics (e.g., audit and feedback meetings) [36] or drug prescribing (e.g., educational outreach visits) [22] in two studies. The other included studies provided no active strategies, passive dissemination of guidelines, or implementation materials to the control group at the end of the study [23, 29–35].

### Guideline implementation strategies

A variety of guideline implementation strategies were used in the included studies. Many combined more than two strategies to implement CPGs. These included educational outreach visits [22, 29–31, 36], teaching sessions [32, 35], seminars [34], audit and feedback [23, 36], and reminders [29, 35]. Printed materials were provided in eight studies [21, 29–35]. Videos were utilized in two included studies to demonstrate practice-based examples, barriers, and solutions [30] and hand hygiene techniques [35]. After the first visit or teaching session, feedback of the performance was provided to the healthcare professionals in four included studies [22, 23, 33, 36]. For the duration of the guideline implementation strategies, educational outreach visits, teaching sessions, and seminars were 15 to 120 min in duration and were conducted in one to four sessions over one to six months [22, 23, 29–36]. Two studies utilized theories to develop their implementation strategies, such as the Social Cognitive Theory and the Health Action Process Approach [30, 32]. Consequently, no study applied an implementation framework or a sustainability-related theoretical framework.

### Sustainability evaluation methods

None of the included studies utilized purpose-designed measurements or structural methods to evaluate sustainability levels. The most common strategy to evaluate sustainability was the analysis of routinely collected data from healthcare professionals. For example, drug prescribing was used in seven included studies [21–23, 30, 31, 33, 36]. Sustainability outcomes included guideline-recommended behaviours (e.g., blood pressure and glycaemic control prescribing and physical activity and nutrition advice) [30], disease management quality indicator adherence (e.g., referral for physical therapy, evaluation of retinopathy) [29, 34], cancer screening compliance [32], and hand hygiene compliance [35]. Seven included studies collected sustainability outcomes at multiple primary care centres using a cluster RCT design [23, 30–34, 36].

### Methodological quality and risk of bias of the included studies

Table 3 illustrates the risk of bias summary of each included study. Only 3 studies fulfilled six of the nine methodological quality criteria and were rated as having a relatively lower risk of bias (a higher score represented a lower risk of bias) [30, 31, 36], Seven studies fulfilled four or fewer criteria [21, 23, 29, 32–35]. Common forms of potential bias across all studies included a limited description of the approach of randomization, allocation concealment, missing data, blind outcome assessment, and protection against contamination. For example, although randomization was mentioned in nine RCTs, only four described the precise information of the random sequence generation process (low risk of bias) [22, 30, 31, 36]. Adequate allocation concealment was reported in three of these RCTs (low risk of bias) [30, 31, 36]. Limited information was used in the evaluation of the risk of bias in two studies as these two studies were published as a brief research report [21, 23]. Three RCTs described and utilized intention to treat analysis (low risk of attrition bias) [31, 32, 35]. Critically, funnel plot analysis was not feasible in this review because of the scarcity of included studies.

**Table 3:** Methodological quality assessment of included studies

	Studies	Item 1	Item 2	Item 3	Item 4	Item 5	Item 6	Item 7	Item 8	Item 9
**1**	Spitaels D, 2019, Belgium [29]	High	Unclear	Low	High	Low	Unclear	Unclear	Low	Unclear
**2**	Presseau J, 2018, England [30]	Low	Low	Low	Low	Low	Low	Low	Low	High
**3**	Pinto D, 2018, Portugal [31]	Low	Low	Low	Low	Low	High	High	Low	High
**4**	Wang H-YJ, 2018, USA [32]	Unclear	Unclear	Low	Low	Low	Unclear	Unclear	Low	Unclear
**5**	Trietsch J, 2017, Netherlands [36]	Low	Low	Low	Low	Low	Low	High	Low	Unclear
**6**	van der Velden AW, 2017, Netherlands [33]	Unclear	High	Low	Low	Unclear	Unclear	Unclear	Low	Unclear
**7**	Noto H, 2016, Japan [34]	Unclear	Unclear	Low	High	Unclear	Low	Low	Low	High
**8**	Gerber JS, 2014, USA [23]	Unclear	Unclear	Unclear	Unclear	Unclear	Unclear	Unclear	Unclear	Unclear
**9**	Martín-Madrazo C, 2012, Spain [35]	Unclear	Unclear	Low	Low	Unclear	Unclear	Unclear	Low	Unclear
**10**	Enriquez-Puga A, 2009, England [22]	Low	Unclear	Low	Low	Unclear	Low	Unclear	Low	Unclear
**11**	Cates CJ 2009, England [21]	High	High	High	High	Unclear	Unclear	Unclear	Low	Unclear

Item 1 random sequence generation; Item 2 adequate concealment of allocation; Item 3 similar baseline outcome measures; Item 4 similar baseline characteristics; Item 5 blinding of outcome assessment; Item 6 adequately addressed incomplete outcome data; Item 7 adequate protection against contamination; Item 8 free from selective reporting; and Item 9 free of other risk of bias

Source: *https://epoc.cochrane.org/resources/epoc-resources-review-authors*

Low = “Low risk”, High = “High risk”, Unclear = “Unclear risk”

### Descriptive analysis of sustainability outcomes

#### Sustainability of drug prescribing improvement

Six studies targeted sustainable improvements in the quality of drug prescribing in daily practice according to the specific recommendations of guidelines in primary care [21–23, 31, 33, 36]. One trial showed no sustained effectiveness differences in drug prescribing in accordance with the guidelines between the intervention and the control groups at 18 months after the intervention (e.g., proportion of COX-2 inhibitors prescribed: 12.07% vs 13.08%, *P*  =  0.085; proportion of omeprazole prescribed: 46.28% vs 47.15%, *P*  =  0.971) [31]. Trietsch et al. utilized audit and feedback for three different practice topics, and the increase in inappropriate testing and prescribing behaviour was 20% in the intervention group and 66% in the control group at nine months after the meeting [36]. Overall, the study did not show a decrease in the volume of inappropriate test ordering and drug prescribing after nine months of the intervention; however, a lesser increase was found in the intervention group [36].

Another study [22] aimed to improve the prescribing of selected antibiotics and antidepressants and reported that educational outreach visits showed no effect on prescribing quality, except that the prescribing of lofepramine (a tricyclic antidepressant) had increased. Educational outreach visits had a small, sustained effect on drug prescribing over the 24-month follow-up period, and prescribing lofepramine increased according to the guidelines, with the rate ratio = 2.85 (*P*  < 0.001) [22]. With the aim of achieving sustainable effectiveness in antibiotic prescribing quality, van der Velden et al. [33] adopted a multifaceted implementation programme and reported significant improved antibiotic prescribing quality between the two groups in dispensed antibiotics per 1000 patients one year (− 7.6% vs − 0.4%, *P*  =  0.002) and two years (− 4.3% vs + 2.0%, *P* =  0.015) after the intervention (decreased prescription indicates improvement). Similar results were reported for macrolides and amoxicillin/clavulanate prescribing, with the first year − 12.7% vs + 2.9% (*P*  =  0.001) and the second year − 7.8% vs + 6.7% (*P*  =  0.005) after the intervention [33]. Van der Velden et al. concluded that part of the multifaceted programme improvement was sustainable, as changes between the two groups were still present 24 months after the intervention [33]. During three-year follow-up, evidence-based patient handouts brought about a sustainable reduction in antibiotics prescribing for patients with acute otitis media [21]. Gerber et al. [23] reported similar results, as antibiotic prescribing decreased from 26.8 to 14.3% in the intervention group and decreased from 28.4 to 22.6% in the control group at 18 months after the intervention.

#### Sustainability of chronic disease management

Three studies aimed to improve the community management of diabetes [30, 34] and knee osteoarthritis [29]. Noto et al. conducted an RCT with an intervention arm that additionally provided a copy of *The Standard Diabetes Manual* and a 30-min seminar regarding *The Standard Diabetes Manual* compared with the control group, which received a copy of *Diabetes Treatment Guide* only [34]. The proportion of GPs who adhered to urinary albumin excretion measurement was significantly higher in the intervention arm (17.9%) compared with the control arm (5.3%) over a 12-month follow-up period (*P* = 0.016) [34]. Another study developed an intervention aimed at enhancing six guideline-recommended healthcare professional behaviours in type 2 diabetes management. Unfortunately, this intervention did not offer a significant improvement in any of these six behaviours at 12 months follow-up [30]. However, about 80% of the patients were examined for circulation and sensation in their feet, more than 70% of patients with a BMI > 30 were provided personalized nutrition advice, and about 50% of the patients were prescribed additional therapy for blood pressure and personalized physical activity advice at 12 months follow-up. Overall, these behaviours’ sustainability level was relatively high during the follow-up period [30]. In addition, educational outreach visits did not lead to significant changes in adherence to quality indicators for knee osteoarthritis management (e.g., referral for physical therapy) among GPs at six months after the outreach visits (43.8% vs 31.3%, *P*  =  0.057) [29].

#### Sustainability of cancer screening

One cluster RCT evaluated an intervention targeted at training GPs to increase patients’ colorectal cancer screening in primary care, and the intervention consisted of three components: a printed communication guide; two structured training sessions; and auxiliary materials [32]. At 12 months follow-up, the colorectal cancer screening rates were slightly higher in the intervention group (24.4%) compared with those in the control group (17.7%), however such difference was not statistically significant (*P* = 0.24) [32].

#### Sustainability of hand hygiene practice

One cluster RCT aimed to test a multimodal hand hygiene improvement programme for healthcare professionals (including assistants in nursing, dental hygienists, GPs, nurses, paediatricians, midwives, and odontostomatologists) for an improved hand hygiene compliance level in primary healthcare centres [35]. The multimodal intervention consisted of the implementation of hydroalcoholic solutions, teaching sessions, and reminder posters [35]. During a six-month follow-up period, the healthcare professionals in the intervention arm enhanced their hand hygiene compliance level by 21.6% compared with the control arm, but the hand hygiene compliance of the healthcare professionals in the intervention group did not significantly improve, and remained at 32.74% [35].

## Discussion

This literature analysis identified 11 studies relevant to this important clinical topic. The duration of follow-up varied from six to 36 months. The results of the included studies showed that guideline implementation strategies (e.g., educational outreach visits, teaching sessions, reminders, and audit and feedback) potentially improve the sustainability of healthcare professionals’ adherence to CPGs in drug prescribing, disease management (e.g., diabetes), and hand hygiene practice in primary care [21, 29, 30, 33, 35]. However, as there was a variety of implementation protocols and outcome measurements reported in the included studies, it was difficult to quantify the extent to which CPG-based healthcare behaviours were sustained, and none of the included studies could be utilized in a meta-analysis. No structured evaluation methods or purpose-designed tools were utilized to assess the healthcare professionals’ sustainability levels. The included studies reported that achieving sustainable adherence to CPGs was a complex goal that was often hampered by practicalities and continued active efforts to sustain improvements [21, 23, 30, 31]. When designing a CPG implementation programme, maximum effort should be taken to ensure the long-term continuation of the implementation by planning the sustainability of adherence to CPGs carefully and adopting a multipronged strategic approach.

One relevant systematic review [8] identified 14 studies that aimed to improve the sustainability of healthcare professionals’ adherence to CPGs in a wide range of medical care settings (acute care and primary care). Three studies included in that review were also considered in this analysis. The published review also reported that long-term adherence (more than one year after implementation) was not sustained in about half of the studies [8]. Moreover, it reported that no firm conclusions about the sustainability of healthcare professionals’ adherence to CPGs in medical practice could be drawn based on the absence of a uniform definition, limited methodological rigour, and the mixed results of the studies [8]. Another systematic integrative review focused on the sustainability of healthcare system improvements, interventions, and programmes and reported that the body of literature was limited, with inconsistent definitions and measures of sustainability [37]. The findings of our literature analysis were consistent with these previous reviews.

According to the findings of our literature analysis, we concluded that the sustainability of healthcare professionals’ adherence to CPGs in primary care was unsatisfactory, and that knowledge about structured approaches to sustaining adherence to CPGs among primary care professionals remains limited. Moreover, only two studies used theories or theoretical frameworks to design their implementation programmes [30, 32]. Healthcare professionals should be guided by suitable frameworks, models and theories (F/M/Ts) to advance sustainability in healthcare with an understanding of the factors that contribute to sustainability [38, 39]. Different F/M/Ts have been used for establishing the theoretical based strategies to facilitate and sustain implementation programs [40], such as Dynamic Sustainability Framework (DSF) and NHS Sustainability Model (NHS SM) and Sustaining Organizational Change Framework (SOCF) [41]. The goals of the use of F/M/Ts in implementation programs including guiding the process of transferring updated evidence into healthcare practice, explaining influencing factors of implementation outcomes and evaluating implementation [42].

Conceptual and methodological limitations in measuring the outcomes of sustainability have been found. A related challenge is that conceptual frameworks with clear operational definitions or rigorous measures of sustainability are not often used [43], as was the case in most of the included studies. Measuring the sustainability of healthcare professionals’ adherence to CPGs is complex, with relatively little attention paid to long-term maintenance [44]. Moore et al. described five elements for the assessment of sustainability, including a defined period of time and the continued delivery of an intervention and/or maintenance of beneficial behaviour, meaning behavioural changes may evolve or adapt while continuing to produce benefits [45]. Other common terms and concepts covered by these studies included durability [23], persistence [31], follow-up [22, 30–32, 34, 36], and long term [31]. Trial evaluation periods provided clear, final evaluation timepoints, ranging from six months [29, 35] to the longest evaluations reported at three years [21]. The evaluation methods to assess sustainability levels of CPG adherence differed across the included studies, and none utilized validated purpose-designed tools. The most common form of evaluation was the analysis of routinely collected data from healthcare professionals.

### Quality of the evidence

Overall, we found a limited body of evidence for any given guideline implementation strategy. The included studies did not provide sufficient evidence to determine the effectiveness of the interventions for improving the sustainability of healthcare professionals’ adherence to CPGs in primary care. Less than one in three studies were rated as having a relatively lower risk of bias, so the levels of evidence in majority of the studies were downgraded because of the significant risk of bias. Seven studies used a cluster design, and the unit of randomization was the cluster (e.g., primary care centres), not the individual healthcare professional, so the baseline characteristics of the healthcare professionals were not comparable [22, 30, 32–36]. Moreover, none of the identified studies used validated purpose-designed tools to measure sustainability levels, and the most common evaluation approach used to measure sustainability was the analysis of routinely collected data from healthcare professionals. In addition, only six studies performed a sample size calculation to justify the included sample [29–31, 33, 35, 36]. This literature analysis found a serious risk of bias; moreover, the small number of studies for each intervention and heterogeneous outcomes prevented us from drawing definitive conclusions.

### Implications for further research and practice

Based on this synthesis of the relevant literature, there is a clear indication for more rigorous studies to develop guideline implementation strategies to improve the sustainability of healthcare professionals’ adherence to CPGs in primary care. While one or two included studies evaluated similar interventions and similar guidelines, we found insufficient data on which to base a conclusion on the most effective approaches or recommendations to address this key clinical issue. This includes identifying the key enablers and barriers to maintain the application of CPGs in the primary care setting. No included studies were identified from low-income countries. While this may be attributable to a relatively lower prominence of primary care in different healthcare systems, as well as the resource-intensive nature of designing and conducting implementation studies in low- and middle-income countries [15, 46] this is represents a key priority for future research.

The core implementation programmes of all the included studies included some type of educational approach (e.g., educational outreach visits, teaching sessions, and written materials) and reported that part of the effect was sustainable [21, 29, 30, 33, 35]. Even if educational strategies are an important element in the sustained process, theory-based implementation interventions may be worth further research, and behavioural change techniques or behavioural change models may help guide the development of these interventions. The standard definition and use of F/M/Ts should be developed to guide implementation program design to facilitate program long-term sustainability. Understanding the determinants of the sustainability of healthcare professionals’ adherence to CPGs and including elements to address them may facilitate sustained benefits in healthcare services and patient outcomes over time. Moreover, as all the included studies relied on self-reports to assess the sustainability outcomes, advancing the measurement of sustainability outcomes through robust prospective designs and using the validated purpose designed sustainability assessment tools are also critical.

The longer-term sustainability of CPG adherence (e.g., two or more years after implementation) warrants further investigation. The appropriate timeframe depends on the nature of the implementation guidelines and on what is relevant for the health behaviours studied. A timeframe that is beyond the initial improvement period to provide meaningful data must be chosen when exploring sustainability interventions [47]. Where a long term change is desirable, assessing sustainability longitudinally over several years is essential to capture variations over time (e.g., conceptualizing the dynamic and nonlinear nature of sustainability) [43]. In addition, most of the included studies aimed to test the effectiveness of the initial implementation innovations without a planned long-term sustainability assessment. Therefore, more studies that include long-term sustainability programs with rigorous multi-component measures of sustainability are needed, implementation program is meaningful only if program results can be sustained.

## Conclusion

This study advanced the understanding that some implementation strategies may potentially improve the sustainability of healthcare professionals’ adherence to CPGs in primary care. Critically, this remains low and unsatisfactory; thereby reducing the potential benefits and impact of CPGs to primary care patients. None of the identified studies applied validated purpose-designed tools to evaluate the sustainability of healthcare professionals’ adherence to CPGs. Consequently, there is a great need to develop theory-based or framework-driven interventions with further rigorous research aims to improve this important indicator of evidence-based practice.

## Supplementary Information

**Additional file 1: Supplemental file 1**. Suggested risk of bias criteria for EPOC reviews.

**Additional file 2.** Searching Strategies.

## Data Availability

All data generated or analysed during this study are included in this published article.

## Abbreviations

CPGs: Clinical practice guidelines
PCPs: Primary care providers
RCT: Randomized controlled trial
EPOC: Effective practice and organization of care
