# Supplementary material for: Sustainability of healthcare professionals’ adherence to clinical practice guidelines in primary care
Source: BMC Prim Care. 2022 Mar 1;23:36. doi: 10.1186/s12875-022-01641-x (PMC8889781; doi:10.1186/s12875-022-01641-x)
Supplement: Supplementary file 2 — Additional file 2. Searching Strategies. [file 12875_2022_1641_MOESM2_ESM.docx]

**Appendix 1. Searching Strategies**

| **Items** | **Searching Strategy** | **Results** |
| --- | --- | --- |
| **PubMed** | | |
| #1 | ((((((Guideline Adherence[MeSH Terms]) OR (Program Sustaina*[Title/Abstract])) OR (Sustainab*[Title/Abstract])) OR (Sustain*[Title/Abstract])) OR (Adherence*[Title/Abstract])) OR (Compliance*[Title/Abstract])) OR (Maintenance*[Title/Abstract]) | [905,712](https://pubmed.ncbi.nlm.nih.gov/?term=%28%28%28%28%28%28Implementation+Science%5BMeSH+Terms%5D%29+OR+%28Program+Sustainabilit%2A%5BTitle%2FAbstract%5D%29%29+OR+%28Sustainabilit%2A%5BTitle%2FAbstract%5D%29%29+OR+%28Sustain%2A%5BTitle%2FAbstract%5D%29%29+OR+%28Adherence%2A%5BTitle%2FAbstract%5D%29%29+OR+%28Compliance%2A%5BTitle%2FAbstract%5D%29%29+OR+%28Maintenance%2A%5BTitle%2FAbstract%5D%29&sort=relevance) |
| #2 | (((((((((Practice Guidelines as Topic[MeSH Terms]) OR (Guidelines as Topic[MeSH Terms])) OR (Health Planning Guidelines[MeSH Terms])) OR (Implementation Science[MeSH Terms])) OR (Guideline*[Title/Abstract])) OR (pathway*[Title/Abstract]))) OR (Consensus[MeSH Terms])) OR (Evidence-based Recommendation*[Title/Abstract])) OR (expert opinion*[Title/Abstract]) | 1,703,726 |
| #3 | #1 AND #2 | 113,648 |
| #4 | (((((Primary Health Care[MeSH Terms]) OR (Primary Care Nursing[MeSH Terms])) OR (Community Health Services[MeSH Terms])) OR (Primary care[Title/Abstract])) OR (Primary healthcare[Title/Abstract])) OR (Community healthcare[Title/Abstract]) | 531,684 |
| #5 | #3 AND # 4 | 7,683 |
| #6 | ((((((((((Health Personnel[MeSH Terms]) OR (general practitioner[MeSH Terms])) OR (Nurses, Community Health[MeSH Terms])) OR (allied health provider*[Title/Abstract])) OR (community healthcare worker*[Title/Abstract])) OR (Healthcare professional*[Title/Abstract])) OR (Health professional*[Title/Abstract])) OR (Allied Health Professional*[Title/Abstract])) OR (Therapist*[Title/Abstract])) OR (Dietitian*[Title/Abstract])) OR (Paramedics[Title/Abstract]) | 644,930 |
| #7 | # 5 AND #6 | 1,715 |
| #8 | Filters applied: Clinical Trial, Humans, from 2000/1/1 - 2021/5/4. | **173** |
| **Cochrane Central Register of Controlled Trials (CENTRAL)** | | |
| #1 | MeSH descriptor: [Guideline Adherence] explode all trees | 1,073 |
| #2 | (Sustainab*):ti,ab,kw OR (Sustain*):ti,ab,kw OR (Adherence*):ti,ab,kw OR (Compliance*):ti,ab,kw OR (Maintenance*):ti,ab,kw | 150,245 |
| #3 | #1 OR #2 | 150,245 |
| #4 | MeSH descriptor: [Practice Guidelines as Topic] explode all trees | 1,635 |
| #5 | MeSH descriptor: [Guidelines as Topic] explode all trees | 1,920 |
| #6 | MeSH descriptor: [Health Planning Guidelines] explode all trees | 13 |
| #7 | (Guideline*):ti,ab,kw OR (Consensus*):ti,ab,kw OR ("Evidence-based Recommendation*"):ti,ab,kw (Word variations have been searched) | 50,030 |
| #8 | #4 OR #5 OR #6 OR #7 | 50,030 |
| #9 | #3 AND #8 | 10,223 |
| #10 | ("Primary Health Care"):ti,ab,kw OR ("Primary Care Nursing"):ti,ab,kw OR ("Community Health Services"):ti,ab,kw OR ("Primary Care"):ti,ab,kw OR ("Primary Healthcare"):ti,ab,kw (Word variations have been searched) | 22,481 |
| #11 | #9 AND #10 | 1,207 |
| #12 | MeSH descriptor: [Health Personnel] explode all trees | 9,150 |
| #13 | MeSH descriptor: [General Practitioners] explode all trees | 282 |
| #14 | MeSH descriptor: [Nurses, Community Health] explode all trees | 23 |
| #15 | MeSH descriptor: [Allied Health Personnel] explode all trees | 1,205 |
| #16 | ("Allied health provider*"):ti,ab,kw OR ("Health professional*"):ti,ab,kw OR ("Health Personnel"):ti,ab,kw OR ("Community healthcare worker*"):ti,ab,kw OR ("Healthcare professional*"):ti,ab,kw (Word variations have been searched) | 9,067 |
| #17 | ("Allied health provider*"):ti,ab,kw OR ("Health professional*"):ti,ab,kw OR ("Health Personnel"):ti,ab,kw OR ("Community healthcare worker*"):ti,ab,kw OR ("Healthcare professional*"):ti,ab,kw (Word variations have been searched) | 14,560 |
| #18 | #12 OR #13 OR #14 OR #15 OR #16 OR #17 | 29,585 |
| #19 | #11 AND #18 with Cochrane Library publication date from Jan 2000 to May 2021, in Trials | **227** |
| **Web of Science** | | |
| #1 | TS=("Guideline Adherence") OR TS=(Sustainab*) OR TS=(Sustain*) OR TS=(Adherence*) OR TS=(Compliance*) OR TS=(Maintenance*)  Indexes=SCI-EXPANDED, SSCI, A&HCI, CPCI-S, CPCI-SSH, ESCI, CCR-EXPANDED, IC Timespan=All years | [1,579,521](https://apps.webofknowledge.com/summary.do?product=WOS&doc=1&qid=8&SID=F1BEWmmbVlauLPXljBW&search_mode=AdvancedSearch&update_back2search_link_param=yes) |
| #2 | TS=(Guideline*) OR TS=("Implementation Science") OR TS=(Pathway*) OR TS=(Consensus) OR TS=("Evidence-based Recommendation*") OR TS=("Expert opinion*")  Indexes=SCI-EXPANDED, SSCI, A&HCI, CPCI-S, CPCI-SSH, ESCI, CCR-EXPANDED, IC Timespan=All years | [2,318,025](https://apps.webofknowledge.com/summary.do?product=WOS&doc=1&qid=12&SID=F1BEWmmbVlauLPXljBW&search_mode=AdvancedSearch&update_back2search_link_param=yes) |
| #3 | #2 AND #1 | [145,161](https://apps.webofknowledge.com/summary.do?product=WOS&doc=1&qid=13&SID=F1BEWmmbVlauLPXljBW&search_mode=CombineSearches&update_back2search_link_param=yes) |
| #4 | TS=("Primary Health Care") OR TS=("Community Health Service") OR TS=("Primary care") OR TS=("Primary healthcare") OR TS=("Community healthcare")  Indexes=SCI-EXPANDED, SSCI, A&HCI, CPCI-S, CPCI-SSH, ESCI, CCR-EXPANDED, IC Timespan=All years | [161,979](https://apps.webofknowledge.com/summary.do?product=WOS&doc=1&qid=16&SID=F1BEWmmbVlauLPXljBW&search_mode=AdvancedSearch&update_back2search_link_param=yes) |
| #5 | #4 AND #3 | [4,666](https://apps.webofknowledge.com/summary.do?product=WOS&doc=1&qid=17&SID=F1BEWmmbVlauLPXljBW&search_mode=CombineSearches&update_back2search_link_param=yes) |
| #6 | TS=("Health Personnel") OR TS=("General practitioner") OR TS=("Allied health provider") OR TS=("Community Nurse") OR TS=("Community healthcare worker") OR TS=("Healthcare professional") OR TS=("Health professional") OR TS=(Therapist) OR TS=(Dietitian) OR TS=(Paramedics)  Indexes=SCI-EXPANDED, SSCI, A&HCI, CPCI-S, CPCI-SSH, ESCI, CCR-EXPANDED, IC Timespan=All years | [86,560](https://apps.webofknowledge.com/summary.do?product=WOS&doc=1&qid=19&SID=F1BEWmmbVlauLPXljBW&search_mode=AdvancedSearch&update_back2search_link_param=yes) |
| #7 | (#6 AND #5) AND LANGUAGE: (English) AND DOCUMENT TYPES: (Article)  Indexes=SCI-EXPANDED, SSCI, A&HCI, CPCI-S, CPCI-SSH, ESCI, CCR-EXPANDED, IC Timespan=2000-2021 | [**210**](https://apps.webofknowledge.com/summary.do?product=WOS&doc=1&qid=22&SID=F1BEWmmbVlauLPXljBW&search_mode=AdvancedSearch&update_back2search_link_param=yes) |
| **EBSCOhost: Medline + Cumulative Index to Nursing and Allied Health Literature (CINAHL) + PsycoINFO** | | |
| #1 | TI Sustainab* OR AB Sustainab* OR TI Sustain* OR AB Sustain* OR TI Adherence* OR AB Adherence* OR TI Compliance* OR AB Compliance* OR TI Maintenance* OR AB Maintenance* OR TI "Guideline Adherence" OR AB "Guideline Adherence" | 1,272,890 |
| #2 | TI Guideline* OR AB Guideline* OR TI "Implementation Science*" OR AB "Implementation Science*" OR TI Pathway* OR AB Pathway* OR TI Consensus OR AB Consensus OR TI "Evidence-based Recommendation*" OR AB "Evidence-based Recommendation*" OR TI "expert opinion*" OR AB "expert opinion*" | 2,194,693 |
| #3 | #2 AND #1 | 135,543 |
| #4 | TI "Primary Care" OR AB "Primary Care" OR TI "Primary Health Care" OR AB "Primary Health Care" OR TI "Community healthcare" OR AB "Community healthcare" OR TI "Primary healthcare" OR AB "Primary healthcare" OR TI "Community Health Service*" OR AB "Community Health Service*" | 265,672 |
| #5 | #4 AND #3 | 5,298 |
| #6 | TI "Health Personnel" OR AB "Health Personnel" OR TI "Health professional*" OR AB "Health professional*" OR TI "general practitioner" OR AB "general practitioner" OR TI "Community nurse" OR AB "Community nurse" OR TI Therapist* OR AB Therapist* OR TI Dietitian* OR AB Dietitian* | 342,681 |
| #7 | #6 AND #5 Limiters - Published Date: 20000101-20210531; English Language  (MEDLINE: 178; CINAHL: 115; PsycInfo: 32) | **325** |
| **Embase + Joanna Briggs Institute + Journals@Ovid** | | |
| #1 | "Sustainab*".m_titl. | 21,036 |
| #2 | "Guideline Adherence* ".m_titl. | 877 |
| #3 | "Sustain* ".m_titl. | 73,869 |
| #4 | "Adherence*".m_titl. | 65,808 |
| #5 | "Compliance*".m_titl. | 40,070 |
| #6 | "Maintenance*".m_titl. | 67,060 |
| #7 | #1 or #2 or #3 or #4 or #5 or #6 | 245,361 |
| #8 | "Primary Health Care".m_titl. | 11,557 |
| #9 | "Primary Care Nursing".m_titl. | 124 |
| #10 | "Community Health Service*".mp. [mp=ti, ab, tx, ct, hw, sw, tn, ot, dm, mf, dv, kw, fx, dq] | 6,325 |
| #11 | "Primary care".mp. [mp=ti, ab, tx, ct, hw, sw, tn, ot, dm, mf, dv, kw, fx, dq] | 438,186 |
| #12 | "Primary healthcare".mp. [mp=ti, ab, tx, ct, hw, sw, tn, ot, dm, mf, dv, kw, fx, dq] | 20,755 |
| #13 | "Primary healthcare".mp. [mp=ti, ab, tx, ct, hw, sw, tn, ot, dm, mf, dv, kw, fx, dq] | 5,463 |
| #14 | #8 or #9 or #10 or #11 or #12 or #13 | 467,267 |
| #15 | #7 and #14 | 6,869 |
| #16 | "Health Personnel".mp. [mp=ti, ab, tx, ct, hw, sw, tn, ot, dm, mf, dv, kw, fx, dq] | 9,8564 |
| #17 | "General practitioner".mp. [mp=ti, ab, tx, ct, hw, sw, tn, ot, dm, mf, dv, kw, fx, dq] | 174,252 |
| #18 | "Community Nurse*".mp. [mp=ti, ab, tx, ct, hw, sw, tn, ot, dm, mf, dv, kw, fx, dq] | 11,189 |
| #19 | "Allied health provider*".mp. [mp=ti, ab, tx, ct, hw, sw, tn, ot, dm, mf, dv, kw, fx, dq] | 570 |
| #20 | "Community healthcare worker*".mp. [mp=ti, ab, tx, ct, hw, sw, tn, ot, dm, mf, dv, kw, fx, dq] | 338 |
| #21 | "Healthcare professional*".m_titl. | 6,334 |
| #22 | "Health professional*".m_titl. | 12,435 |
| #23 | Therapist*.m_titl. | 14,682 |
| #24 | Dietitian*.m_titl. | 2,676 |
| #25 | "Paramedics".m_titl. | 1,738 |
| #26 | #16 or #17 or #18 or #19 or #20 or #21 or #22 or #23 or #24 or #25 | 314,140 |
| #27 | #15 and #26 | 912 |
| #28 | limit 27 to clinical trial [Limit not valid in Journals@Ovid,JBI EBP Database; records were retained] | 295 |
| #29 | limit 28 to english language [Limit not valid in Journals@Ovid,JBI EBP Database; records were retained] | 295 |
| #30 | limit 29 to original articles [Limit not valid in JBI EBP Database,Embase; records were retained] | 229 |
| #31 | limit 30 to yr="2000 - 2021" | **215** |
